# Supplementary material for: Exon Targeted Retrieval and Classification Toolbox (ExTRaCT): a gene search pipeline to find APOBEC3 Z-domains in novel bat genomes
Source: bioRxiv. 2026 Apr 14:2026.03.15.711917. Originally published 2026 Mar 18. Preprint. [Version 2] doi: 10.64898/2026.03.15.711917 (PMC13015710; doi:10.64898/2026.03.15.711917)
Supplement: Supplement 1 [file media-1.pdf]

Supplementary Materials (SM)

SM1 Background on Bat Immunity and Small Gene Annotation

Some believe that bats have heightened immunity because the sustained temperature held during flight contributes to positive selection on DNA damage and repair genes linked to enhanced immunity and disease tolerance (Banerjee et al., 2020; O'Shea et al., 2014; Zhang et al., 2013). While this hypothesis is often cited as an explanation for certain increases by gene duplication in bat immunity genes, it does not account for other demographic or behavioral differences that can shape the immune system such as roosting, diet, geographical realm or exposure to disease. Anthropogenic stressors such as habitat loss and food scarcity amplify bat originating spillover, posing a zoonotic risk to humans (Becker et al., 2023; Carlson et al., 2025; Eby et al., 2023). Our work is motivated by the question of whether variation in A3 Z-domain counts impact bat immunity.

The first step to answer this question through phylogenetic, structural and functional analysis requires robust tools to accurately identify gene families in a target genome assembly. Existing automated methods for genome annotation may overlook or misrepresent gene family member targets, may rely on advanced or computationally burdensome techniques and may be limited to reference genomes of fully annotated model organisms. We mention in the man text examples with TOGA and MAKER, but another annotation tool AUGUSTUS (Stanke & Morgenstern, 2005) which has been trained on many species, requires computational expertise. Additionally, TOGA raw output (Perez et al., 2025) can return missing or partial sequences, requiring noncomputational protocols to make up for this deficit. Together or in some combination, these shortcomings can mislead characterization of gene families. To address these limitations, biologists often have their own manual processes to validate gene searches, with varying degrees of automation. Here we provide an easy-to-use pipeline that is fully computational, filling gaps in traditional out-of-the box tools to enable downstream gene family studies.

SM2 Input sequence and genome data

SM2.1. Table. Accession numbers, source and date downloaded

Names, accession numbers or other identifiers and date of download of (1) reference sequence APOBEC3 (from NCBI (NCBI Resource Coordinators, 2016)), (2) target species genomes (from globus (Bat1K 21 Family Group, 2026)) and (3) six bat A3 (from UCSC senckenberg genome browser (UCSC, 2025))

Species	Sequence Name	Source	#Accession or file name	Date downloaded
Macaca mulatta	A3C	NCBI	EU381233.1	11/18/2024
Chlorocebus aethiops	A3C	NCBI	EU381232.1	11/18/2024
Homo sapiens	A3A (v1) Z1	NCBI	NM_145699.4	11/18/2024
Homo sapiens	A3B (v1) Z2-Z1	NCBI	NM_004900.5	11/18/2024
Homo sapiens	A3C Z2	NCBI	NM_014508.3	11/18/2024
Homo sapiens	A3D (v1) Z2-Z2	NCBI	NM_152426.4	11/18/2024
Homo sapiens	A3F (v1) Z2 – Z2	NCBI	NM_145298.6	11/18/2024
Homo sapiens	A3F (cds) Z2 – Z2	NCBI	JF262037.1	11/18/2024
Homo sapiens	A3G (cds) Z2-Z1	NCBI	JF262036.1	11/18/2024
Homo sapiens	A3H (vSV-154) Z3	NCBI	NM_001166004.3	11/18/2024
Bos taurus	A3 Z1	NCBI	EU864534.1	11/18/2024
Bos taurus	A3Z2	NCBI	EU864535.1	11/18/2024
Bos taurus	A3Z3	NCBI	EU864536.1	11/18/2024
Equus caballus	A3 Z2a-Z2b	NCBI	FJ527822.1	11/18/2024
Equus caballus	A3 Z2c-Z2d	NCBI	FJ527823.1	11/18/2024
Equus caballus	A3 Z3 v1	NCBI	FJ532286.1	11/18/2024
Equus caballus	A3 Z1b	NCBI	FJ532287.1	11/18/2024
Equus caballus	A3 Z2e	NCBI	FJ532288.1	11/18/2024
Equus caballus	A3 Z3	NCBI	FJ532289.1	11/18/2024
Felis catus	A3 Cc	NCBI	EU109281.1_cds_ABW83271.1_1	11/18/2024
Felis catus	A3 Ca	NCBI	EU109281.1_cds_ABW83272.1_2	11/18/2024
Felis catus	A3 Cb	NCBI	EU109281.1_cds_ABW83273.1_3	11/18/2024
Felis catus	A3H	NCBI	EU109281.1_cds_ABW83274.1_4	11/18/2024
Felis catus	A3	NCBI	GU097660.1	11/18/2024
Lynx lynx	A3	NCBI	GU097661.1	11/18/2024
Ovis aries	A3 Z1	NCBI	EU864541.1	11/18/2024
Ovis aries	A3 Z2	NCBI	EU864542.1	11/18/2024
Ovis aries	A3 Z3	NCBI	EU864543.1	11/18/2024
Panthera leo bleyenberghi	A3	NCBI	GU097662.1	11/18/2024
Panthera tigris corbetti	A3	NCBI	GU097663.1	11/18/2024
Puma concolor	A3	NCBI	GU097659.1	11/18/2024
Sus scrofa	A3 Z2	NCBI	EU864539.1	11/18/2024
Sus scrofa	A3 Z3	NCBI	EU864540.1	11/18/2024
Sus scrofa	A3 Z2-Z3	NCBI	EU871586.1	11/18/2024
Sus scrofa	A3 Z2_1	NCBI	FJ716801.2	11/18/2024
Molossus molossus	A3A	UCSC	HLmolMol2 APOBEC3A	10/17/2024
Molossus molossus	A3A2	UCSC	HLmolMol2 APOBEC3A2	10/17/2024
Molossus molossus	A3C	UCSC	HLmolMol2 APOBEC3C	10/17/2024
Molossus molossus	A3H	UCSC	HLmolMol2 APOBEC3H	10/17/2024
Myotis myotis	A3A	UCSC	HLmyoMyo6 APOBEC3A	10/17/2024

Myotis myotis	A3A2	UCSC	HLmyoMyo6 APOBEC3A2	10/17/2024
Myotis myotis	A3A3	UCSC	HLmyoMyo6 APOBEC3A3	10/17/2024
Myotis myotis	A3C	UCSC	HLmyoMyo6 APOBEC3C2	10/17/2024
Myotis myotis	A3C2	UCSC	HLmyoMyo6 APOBEC3C2	10/17/2024
Pipistrellus kuhlii	A3A	UCSC	HLpipKuh2 APOBEC3A	10/17/2024
Pipistrellus kuhlii	A3A2	UCSC	HLpipKuh2 APOBEC3A2	10/17/2024
Pipistrellus kuhlii	A3A3	UCSC	HLpipKuh2 APOBEC3A3	10/17/2024
Pipistrellus kuhlii	A3A4	UCSC	HLpipKuh2 APOBEC3A4	10/17/2024
Pipistrellus kuhlii	A3A6	UCSC	HLpipKuh2 APOBEC3A6	10/17/2024
Pipistrellus kuhlii	A3C	UCSC	HLpipKuh2 APOBEC3C	10/17/2024
Pipistrellus kuhlii	A3C2	UCSC	HLpipKuh2 APOBEC3C2	10/17/2024
Pipistrellus kuhlii	A3C3	UCSC	HLpipKuh2 APOBEC3C3	10/17/2024
Phyllostomus discolor	A3A	UCSC	HLphyDis3 APOBEC3A	10/17/2024
Phyllostomus discolor	A3A2	UCSC	HLphyDis3 APOBEC3A2	10/17/2024
Phyllostomus discolor	A3C2	UCSC	HLphyDis3 APOBEC3C2	10/17/2024
Rousettus aegyptiacus	A3	UCSC	HLrouAeg4 APOBEC3A	10/17/2024
Rousettus aegyptiacus	A3A	UCSC	HLrouAeg4 APOBEC3A	10/17/2024
Rhinolophus ferrumequinum	A3A	UCSC	HLrhiFer5 APOBEC3A	10/17/2024
Rhinolophus ferrumequinum	A3A2	UCSC	HLrhiFer5 APOBEC3A2	10/17/2024
Rhinolophus ferrumequinum	A3B	UCSC	HLrhiFer5 APOBEC3B	10/17/2024
Rhinolophus ferrumequinum	A3C	UCSC	HLrhiFer5 APOBEC3A	10/17/2024
Cistugo seabrae	genome	Bat 1k	mCisSea1.1.hap1	10/18/2024
Craseonycteris thonglongyai	genome	Bat 1k	mCraTho2.1.hap1	10/19/2024
Rhynchonycteris naso	genome	Bat 1k	mRhyNas1.2.hap1	10/18/2024
Saccopteryx bilineata	genome	Bat 1k	mSacBil1.1.pri	10/18/2024
Saccopteryx leptura	genome	Bat 1k	mSacLep1.1.pri	10/18/2024
Taphozous melanopogon	genome	Bat 1k	mTapMel1.1.pri	10/18/2024
Furipterus horrens	genome	Bat 1k	mFurHor1.1.pri	10/18/2024
Aselliscus stoliczkanus	genome	Bat 1k	mAseSto1.2.pri	10/19/2024
Doryrhina cyclops	genome	Bat 1k	mDorCyc1.2.pri	10/19/2024
Hipposideros abae	genome	Bat 1k	mHipAba1.1.hap1	10/19/2024
Hipposideros armiger	genome	Bat 1k	mHipArm2.1.pri	10/19/2024
Hipposideros caffer	genome	Bat 1k	mHipCaf2.1.hap1	10/19/2024
Hipposideros jonesi	genome	Bat 1k	mHipJon1.1.hap1	10/19/2024
Hipposideros larvatus	genome	Bat 1k	mHipLar1.2.pri	10/19/2024
Hipposideros swinhoii	genome	Bat 1k	mHipSwi1.1.pri	10/19/2024
Megaderma spasma	genome	Bat 1k	mMegSpa1.1.pri	10/19/2024
Miniopterus australis	genome	Bat 1k	mMinAus1.1.pri	10/18/2024
Miniopterus natalensis	genome	Bat 1k	mMinNat1.1.hap2	10/18/2024
Miniopterus schreibersii	genome	Bat 1k	mMinSch1.1.hap1	10/18/2024
Eumops nanus	genome	Bat 1k	mEumNan1.1.hap1	10/18/2024
Molossus alvarezi	genome	Bat 1k	mMolAlv1.1.hap1	10/18/2024
Molossus molossus	genome	Bat 1k	mMolMol1.2.pri	10/18/2024
Molossus nigricans	genome	Bat 1k	mMolNig1.2.hap1	10/18/2024
Mops condylurus	genome	Bat 1k	mMopCon1.1	10/19/2024
Tadarida brasiliensis	genome	Bat 1k	mTadBra1.3.pri	10/19/2024
Mormoops megalophylla	genome	Bat 1k	mMorMeg1.1.pri	10/18/2024
Mystacina tuberculata	genome	Bat 1k	mMysTub1.2.pri	10/18/2024
Myzopoda aurita	genome	Bat 1k	mMyzAur1.1.pri	10/18/2024
Natalus tumidirostris	genome	Bat 1k	mNatTum1.1.pri	10/19/2024
Noctilio leporinus	genome	Bat 1k	mNocLep2.1.pri	10/18/2024
Nycteris thebaica	genome	Bat 1k	mNycThe1.1.hap1	10/18/2024
Artibeus intermedius	genome	Bat 1k	mArtIn1.1.hap1	10/18/2024
Artibeus lituratus	genome	Bat 1k	mArtLit1.1.hap1	10/18/2024
Brachyphylla cavernarum	genome	Bat 1k	mBraCav1.1.hap1	10/18/2024
Carollia perspicillata	genome	Bat 1k	mCarPer1.2.pri	10/18/2024
Centurio senex	genome	Bat 1k	mCenSen1.1.hap1	10/18/2024
Choeroniscus minor	genome	Bat 1k	mChoMin1.1.hap1	10/18/2024
Desmodus rotundus	genome	Bat 1k	mDesRot1.15.hap1	10/18/2024
Diaemus youngii	genome	Bat 1k	mDiaYou1.3.hap1	10/18/2024
Diphylla ecaudata	genome	Bat 1k	mDipEca1.2.hap1	10/18/2024
Ectophylla alba	genome	Bat 1k	mEctAlb1.1.hap1	10/18/2024
Erophylla bombifrons	genome	Bat 1k	mEroBom1.1.hap1	10/18/2024
Glossophaga mutica	genome	Bat 1k	mGloMut1.1.hap1	10/18/2024
Glossophaga soricina	genome	Bat 1k	mGloSor1.2.pri	10/18/2024
Glyphonycteris daviesi	genome	Bat 1k	mGlyDav1.1.hap1	10/18/2024

Leptonycteris yerbabuenae	genome	Bat 1k	mLepYer2.1.hap1	10/18/2024
Lionycteris spurrelli	genome	Bat 1k	mLioSpu1.1.hap1	10/18/2024
Lonchorhina inusitata	genome	Bat 1k	mLonInu1.1.hap1	10/18/2024
Macrophyllum macrophyllum	genome	Bat 1k	mMacMac1.1.hap1	10/18/2024
Macrotus waterhousii	genome	Bat 1k	mMacWat1.1.hap1	10/18/2024
Micronycteris megalotis	genome	Bat 1k	mMicMeg1.1.pri	10/18/2024
Phyllostomus discolor	genome	Bat 1k	mPhyDis1.3.pri	10/18/2024
Phyllostomus hastatus	genome	Bat 1k	mPhyHas1.1.pri	10/18/2024
Platyrrhinus guianensis	genome	Bat 1k	mPlaGui1.1.hap1	10/18/2024
Rhinophylla pumilio	genome	Bat 1k	mRhiPum1.1.hap1	10/18/2024
Trachops cirrhosus	genome	Bat 1k	mTraCir3.1.hap1	10/18/2024
Trinycteris nicefori	genome	Bat 1k	mTriNic1.1.hap1	10/18/2024
Uroderma convexum	genome	Bat 1k	mUroCon1.1.pri	10/18/2024
Vampyressa thyone	genome	Bat 1k	mVamThy1.1.hap1	10/18/2024
Cynopterus sphinx	genome	Bat 1k	mCynSph1.1.pri	10/19/2024
Eonycteris spelaea	genome	Bat 1k	mEonSpe1.2.hap1	10/19/2024
Hypsignathus monstrosus	genome	Bat 1k	mHypMon1.1.pri	10/19/2024
Rousettus aegyptiacus	genome	Bat 1k	mRouAeg1.4.pri	10/19/2024
Rhinolophus affinis	genome	Bat 1k	mRhiAff1.2.pri	10/19/2024
Rhinolophus ferrumequinum	genome	Bat 1k	mRhiFer1.5.pri	10/19/2024
Rhinolophus foetidus	genome	Bat 1k	mRhiFoe1.2.pri	10/19/2024
Rhinolophus hipposideros	genome	Bat 1k	mRhiHip1.1.hap1	10/19/2024
Rhinolophus pearsonii	genome	Bat 1k	mRhiPea1.1.pri	10/19/2024
Rhinolophus perniger lanosus	genome	Bat 1k	mRhiPer1.2.pri	10/19/2024
Rhinolophus sinicus	genome	Bat 1k	mRhiSin3.1.pri	10/19/2024
Rhinolophus trifoliatus	genome	Bat 1k	mRhiTri1.2.pri	10/19/2024
Rhinolophus yonghoiseni	genome	Bat 1k	mRhiYon1.2.pri	11/12/2024
Triaenops persicus	genome	Bat 1k	mTriPer1.1.hap1	11/12/2024
Rhinopoma microphyllum	genome	Bat 1k	mRhiMic1.1.pri	11/5/2024
Rhinopoma muscatellum	genome	Bat 1k	mRhiMus1.1.pri	11/12/2024
Thyroptera tricolor	genome	Bat 1k	mThyTri1.1.pri	10/18/2024
Antrozous pallidus	genome	Bat 1k	mAntPal2.1.pri	10/19/2024
Corynorhinus mexicanus	genome	Bat 1k	mCorMex1.1.pri	10/19/2024
Corynorhinus townsendii	genome	Bat 1k	mCorTow1.1.hap1	10/19/2024
Eptesicus fuscus	genome	Bat 1k	mEptFus1.3.pri	10/19/2024
Eptesicus nilssonii	genome	Bat 1k	mEptNil2.1.pri	10/19/2024
Lasiurus ega	genome	Bat 1k	mLasEga1.1.hap1	10/19/2024
Myotis auriculus	genome	Bat 1k	mMyoAur1.1.pri	10/19/2024
Myotis californicus	genome	Bat 1k	mMyoCal1.1.pri	10/19/2024
Myotis daubentonii	genome	Bat 1k	mMyoDau2.1.pri	10/19/2024
Myotis evotis	genome	Bat 1k	mMyoEvo1.1.pri	10/19/2024
Myotis lucifugus	genome	Bat 1k	mMyoLuc2.1.pri	10/19/2024
Myotis myotis	genome	Bat 1k	mMyoMyo1.6.pri	10/19/2024
Myotis mystacinus	genome	Bat 1k	mMyoMys1.1.hap1	10/19/2024
Myotis nigricans	genome	Bat 1k	mMyoNig1.1.pri	10/19/2024
Myotis occultus	genome	Bat 1k	mMyoOcc1.1.pri	10/19/2024
Myotis pilosus	genome	Bat 1k	mMyoPil1.1.pri	10/19/2024
Myotis thysanodes	genome	Bat 1k	mMyoThy1.1.pri	10/19/2024
Myotis velifer	genome	Bat 1k	mMyoVel1.1.pri	10/19/2024
Myotis vivesi	genome	Bat 1k	mMyoViv1.2.pri	10/19/2024
Myotis volans	genome	Bat 1k	mMyoVol1.1.pri	10/19/2024
Myotis yumanensis	genome	Bat 1k	mMyoYum1.1.hap1	10/19/2024
Nyctalus aviator	genome	Bat 1k	mNycAvi1.1.pri	10/19/2024
Pipistrellus kuhlii	genome	Bat 1k	mPipKuh1.2.pri	10/19/2024
Pipistrellus nathusii	genome	Bat 1k	mPipNat1.1.hap1	10/19/2024
Pipistrellus pygmaeus	genome	Bat 1k	mPipPyg1.1.pri	10/19/2024
Plecotus auritus	genome	Bat 1k	mPleAur1.1.pri	10/19/2024
Vespertilio murinus	genome	Bat 1k	mVesMur1.1.pri	10/19/2024

SM3 Architecture Map

SM3.1. Figure. ExTRaCT small gene search pipeline

Pipeline architecture includes search algorithm and post processing steps. Each track corresponds to a custom Python command. The green rounded outline indicates an input file, the green diagonal and rounded outline indicates an output file that may be used as an input for the following track, and the blue squares indicates a process in the code. Preprocessing depends on hmmer and converts a FASTA file into a HMMER profile which is a required input for the gene search algorithm. The core gene search algorithm consists of four major processes with three program dependencies (hmmer, bedtools and getorf) and 3 required input files. The output is a table which can be processed into final FASTA and phylogenetic tree for analysis in the postprocessing tracks.

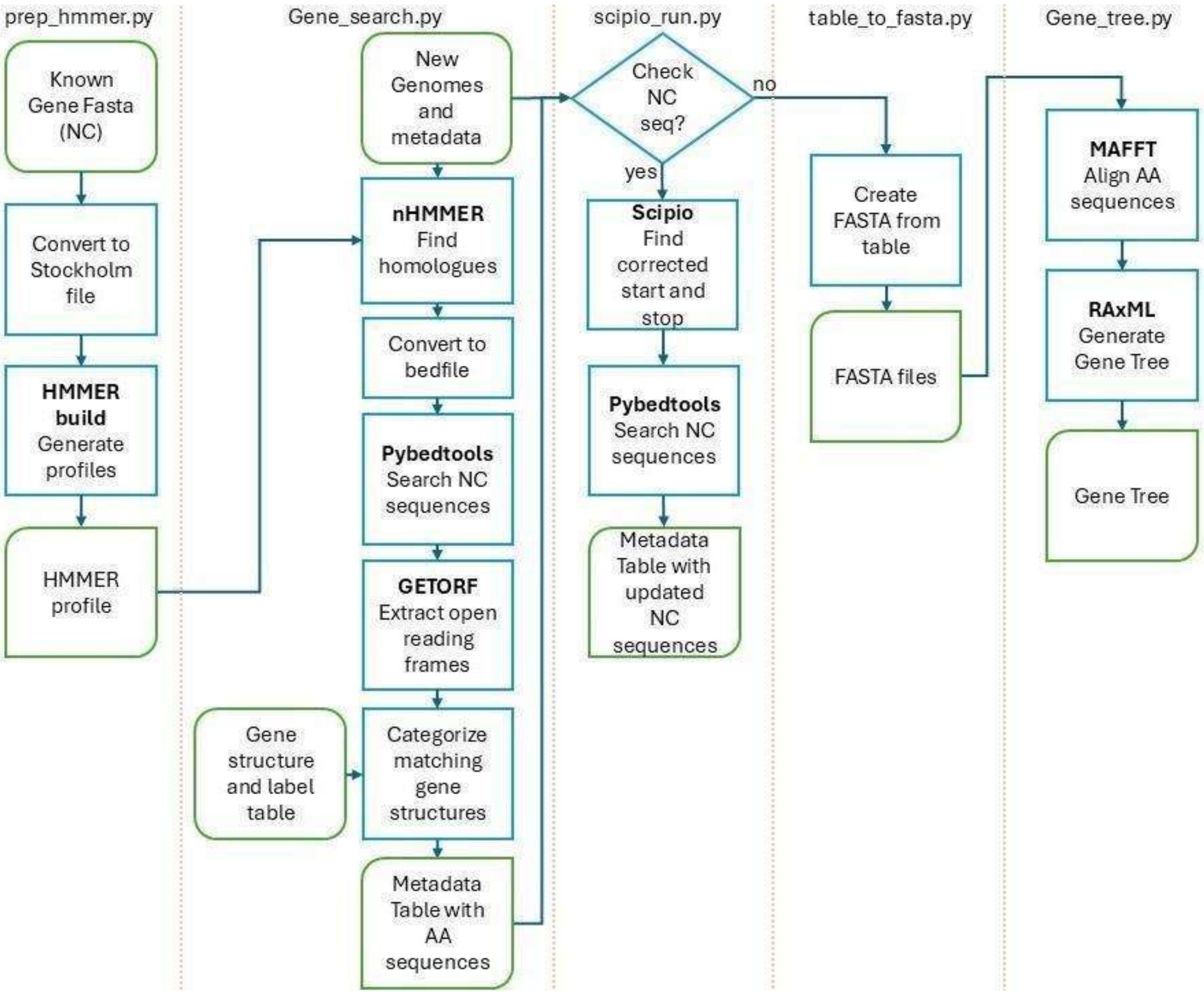

SM3.2. Program requirement details

Python version 3.7.10 (Foundation, 2021) (Anaconda is recommended but not required) Biopython (v1.81) SeqIO and Align IO (Cock et al., 2009) (pandas v.1.3.5 (Reback et al., 2021), regex/re 2024.4.16 (Hoyt, 2024), gzip, sys, os, argparse (Foundation, 2021)), PyBedTools (v 0.10.0) (Dale, Pedersen, & Quinlan, 2011), HMMER version 3.1b2 is required (Eddy, 2011). EMBOSS (v6.6) (Rice, Longden, & Bleasby, 2000), Scipio (v1.4.1) (Keller et al., 2008) (with dependencies in webpage (Scipio, 2025). install BioPerl, Yaml, Blat and the BedOPs function convert2bed), MAFFT (v 7.490) (Katoh & Standley, 2013) with the option "—auto", and RAxML (v8.2.18) (Stamatakis, 2014) with options set to infer trees from amino acid sequence alignments using an automatic search for the best-fit model of protein evolution and a Gamma rate distribution across sites (PROTGAMMAAUTO), keeping identical sequences (--no-seq-check and –print-identical-sequences) to preserve any gene copies, since HMMER returns non overlapping sequences. These settings can be changed easily, as needed. Instructions for installation of dependencies are provided in the help file in the github.

SM4 Input Data Requirements and Test Files

ExTRaCT may be used to search for any conserved single-exon gene or region of interest. ExTRaCT finds genes with known structural motifs and relies on a homolog search program. Four input files are required: the new species genome assembly file(s), a nucleotide homologue sequence FASTA file, a gene protein categorization table and a metadata file. There may be one or many genome assemblies of novel species as targets that have not been annotated or for which coding regions for a certain gene are yet unknown. The FASTA input file with known gene homologues of other species do not need to be in the same taxonomic order or superorder as the target species. The metadata file to initiate the search algorithm requires two columns, the species name and FASTA file path to direct the program. A CSV file with a table of gene labels and corresponding structural motifs is used to filter hits. We show that minor differences in motif definitions greatly impact the count and accuracy of the output.

SM5 Pairwise match inspection for all test scenarios

To determine how homologue search results vary depending on input species we conduct three different tests. The first test considers three known species categories, or taxa: bats, Laurasiatheria and primates. A3s may have 1 or 2 catalytic Z-domains which we refer to as single or double domained A3. The second test checks how the algorithm behaves when single, double or combined single and double domain lists are provided as input homologues. We call this last set "both" and includes all A3 for the given species in the taxa. Domain designation varies across mammals. For the bat A3s from Jebb et al. there are 26 genes: 3 double-domain, 19 single-domain (25 total Z-domains) and 4 missing or partial domain A3s. The set of primate A3s consists of three different species (*Homo sapiens*, *Macaca mulatta*, and *Chlorocebus aethiops*), with a total of 9 A3s, of which 4 are double- and 5 are single-domain. The set of Laurasiatheria A3s span 9 species (*Bos taurus*, *Equus caballus*, *Felis catus*, *Lynx lynx*, *Ovis aries*, *Panthera leo bleyenberghi*, *Panthera tigris corbetti*, *Puma concolor*, and *Sus scrofa*), with a total of 25 A3s, with 8 double- and 17 single-domain. The last test uses three different A3 Z-domain amino acid sequence categories defined by Salter (motif set A) (Salter, Bennett, & Smith, 2016), Hayward (motif set B) (Hayward et al., 2018) and Jebb (motif set C) (Jebb et al., 2020).

Salter was the first to classify human cytidine deaminating Z-domains (zinc-dependent deaminase sequence motif (ZDD) in Activation induced deaminase (AID), APOBEC1, APOBEC2 and APOBEC4), and Z1, Z2 and Z3 in A3). Hayward and Jebb define different bat-specific Z-domain structures. In the table we show the specific changes from motif set A to B (Salter to Hayward), then from motif set B to C (Hayward to Jebb). Hayward identified an additional Z2B domain in pteropodids, but also made four changes to Salter motifs, three of which reduce the motif sequence space. Later, Jebb introduced a Z1B domain in bats and made a combination of restrictive and amplifying changes to the sequence space. For our analysis we refer to the input sequence labels and categories as motif set A for Salter, motif set B for Hayward and motif set C for Jebb. Furthermore, the category motifs we defined explicitly restrict the 9th and 10th position for Z1s by excluding the Tryptophan-Phenylalanine (WF) couplet, so sequences with the WF couplet may be categorized as Z2, reflecting the structure of known bat A3 Z2s. For motif set A we treat the 3 canonical Z-domains in Salter as a total of 6 different motifs wherein each domain may have either a Serine or a Threonine before the PC couplet. Rather than providing further novel definitions of Z-domains in bats we chose to compare and use existing definitions which have been verified through expression analysis (Hayward et al., 2018; Jebb et al., 2020).

SM5.1. Table. Differences between Motif set categories (A, B, C)

Z domain amino acid sequence motif changes between the three test sets of Salter (A),Hayward (B), and Jebb (C). X marks undefined amino acid in position of motif. Red indicates strict restrictions and green expansions of amino acid sequence space. Arrows indicate increase or decrease of number of unspecified amino acid in motif. Blue with an upward arrow indicates an ambiguous change which expands the number of uncharacterized amino acids but does not include sequences captured by the previous motif. Empty squares are regions of the motif with not change from A to B to C.

Label	Motif Category	AA Changes								
Z1	A		SWS	PC		F	RIY		L	
	A		SWT	PC		F	RIY		L	
	B		SWX	PC		F	RIY		L	
	C	↑	SWS	PC		F	RIY		L	
Z1B	C		SWS	PC		F	RIY		L	
Z2	A		SWS	PC		F	RLY		L	
	A		SWT	PC		F	RLY		L	
	B		SWS	PC		F	RLY		L	
	C	↑	SWS	PC		X	RLY		L	↓
Z2B	B		SWS	PC		F	RIY		L	
	C		SWS	PC		F	RIY		L	
Z3	A		TWS	PC		F	RLY		L	
	A		TWT	PC		F	RLY		L	
	B		TWS	PC		F	RLY		L	
	C	↓	TWS	PC		X	RLX		L	

Below are summaries of the 27 test results. We report how the different input tests described, (1) species homologue by clade, (2) gene structure by catalytic arrangement (single domain, double domain and combined single and double domain A3s) and (3) motif filtering definitions (from Salter (Salter, Bennett, & Smith, 2016), Hayward (Hayward et al., 2018) and Jebb (Jebb et al., 2020)), impact results. When relevant, we have listed the amino acid sequences in our three tests below the respective tables.

SM5.2. Table.Test 1: Compare input domain files

The following are mismatched sequences found in the first test which compares different input reference sequences by species clade. The reference input A3 sequences domain type is "both" (list of all A3 sequences) and is tested across all classification motifs.

Sequence key	Species	Found in test result	Missing in test result
Bc1	*Brachyphylla cavernarum*	Laurasitheria-Both-C	Primate-Both-C
Bc1	*Brachyphylla cavernarum*	Chiroptera-Both-C	Primate-Both-C

In this case, for domain type "both" and motif set C, Laurasiatheria and chiroptera have identical results, with a total of 496 matching sequences. The 495 sequences from the primate test results are contained in the 496 sequences, but the primate test is missing a sequence in *Brachuphylla cavernarum*, Bc1, which is present in the Laurasiatheria and chiroptera results. Primates are an order of mammals in the superorder Euarchontoglines, and bats are in the order chiroptera, superorder Laurasiatheria. For species of the same superorder (Laurasiatheria) but different order (not chiroptera), the results are the same.

SM5.3. Table.Test 2: Compare input domain files

The following are mismatched sequences found in the second test which compare different input reference A3 sequences by domain. The three file types are "both" (all single and double domain A3 sequences), "single" (only list of single domain A3 sequences) and "double" (only list of double domain A3 sequences). The reference input sequences tested are across all species clades and are tested across all classification motifs. List of Li1-4 sequences show a single amino acid difference in sequence outputs.

Seq key	Species	Found in test result	Missing in test result
Bc1	*Brachyphylla cavernarum*	Primate-Single-C	Primate-Both-C
Ct 1	*Craseonycteris thonglongyai*	Primate-Both-C	Primate-Single-C
Li 1	*Lonchorhina inusitata*	Primate-Double-A	Primate-Both-A
Li 1	*Lonchorhina inusitata*	Primate-Double-B	Primate-Both-B
Li 2	*Lonchorhina_inusitata*	Primate-Double-A	Primate-Both-A
Li 2	*Lonchorhina_inusitata*	Primate-Double-B	Primate-Both-B
Li 3	*Lonchorhina_inusitata*	Primate-Both-A	Primate-Double-A
Li 3	*Lonchorhina_inusitata*	Primate-Both-B	Primate-Double-B
Li 4	*Lonchorhina_inusitata*	Primate-Both-A	Primate-Double-A
Li 4	*Lonchorhina_inusitata*	Primate-Both-B	Primate-Double-B

Li Sequences
Li1 – HAEQRFLSWFDDTILSRRAEYQVTWYMSWSPCSECAQEVVAFLEAHENVSLSIFASRLYKCEDEDQQQGLRLLDQAKRTEVAM
Li3 – HAEQRFLSWFDDTILSRRAEYQVTWYMSWSPCSECAQEVVAFLEAHENVSLSIFASRLYKCEDEDQQQGLRLLDQAKRTEVAMM
Li2 – HAEQRFLSWFDGTILSRRAEYQVTWYMSWSPCSECAQEVVAFLEVHENVSLSISASRLYKCEDEEQQQGLRLLDQAKRTEVAM
Li4 – HAEQRFLSWFDGTILSRRAEYQVTWYMSWSPCSECAQEVVAFLEVHENVSLSISASRLYKCEDEEQQQGLRLLDQAKRTEVAMM

Combined domain type input results for “both” include sequences from the same single and double tests, with few exceptions. We would expect that while single and double domain inputs may have different results, if structures vary significantly (for example only double domain have a Z3 but none of the single domain have a Z3), that a combined input file with A3s of both domain types, with all Z types, should result in the same sequences as single- and double- domains separately. We found that, for Laurasiatheria and chiroptera, the third option, a combined list of all A3 genes, captures the same results as when single- and double-domain A3 are analyzed separately. For primate species homologues, one or two sequences were found in single- or double-domain searches that were missing in the corresponding combined domain search.

Test 2 table shows the missing sequences in the domain input test. The *Brachyphylla cavernarum* sequence Bc1 is in the result list from the primate, single-domain, motif set C test, but, as we mentioned, not in the primate combined domain motif set C test. The final hit count of primate single and primate combined domain (motif set C) tests are equal because there is a sequence from *Craseonycteris thonglongyai* (Ct1) in the primate combined domain test result missing from the primate single domain test result. There were two sequences found in the primate double motif set A and B test results missing in the primate combined domain (motif set A and B) tests from the species, *Lonchorhina inusitata* (Li1 and Li2). However, in both motif tests, the primate combined domain results list two other distinct sequences, Li3 which matches the missing Li1, and Li4 which matches the missing Li2, with the addition of a methionine at the end for both. The function nhmmer returns nucleotide sequence ends based on a HMMER profile, so it is likely that in the combined domain case, longer sequences hits were generated to find ORFs. In the case of Bc1, the combined HMMER profile is different enough from the single-domain HMMER profile that it completely missed this sequence, even though the single-domain A3s are a subset of the sequences used to generate the combined-domain HMMER profile. One reason the HMMER profiles of the primates are different enough to miss some results is because fewer sequences were used (9 total) compared to those for the Laurasiatheria (25) and chiroptera (26) HMMER profiles. Both examples (missing Bc1 and varied Li1-4 sequences) show that our algorithm is only weakly sensitive to profile differences, and generally using combined domain inputs will capture the appropriate sequences.

It seems that the number of homologous single- or double-domain genes considered for the pipeline input does not impact the number of output sequences, i.e., more single domains does not necessarily yield more results. For example, there are fewer double-domain than single-domain bat A3s, and in our tests double-domain input consistently yielded fewer results than the single-domain input. Yet the primate A3 homologues we selected had more double-domain than single-domain sequences, albeit a smaller overall count of A3s than either the bat homologue or Laurasiatheria homologue files, and still a similar pattern to those seen with the bat homologues arose in primates, for which the double-domain input yielded fewer results than the single-domain input. Laurasiatheria, in contrast, consistently resulted in more sequences for the double-domain test than the single-domain test, although its count of single- and double-domain A3s resembles the bat homologue input. While HMMER is clearly a robust option for homologue search, it is important to test the different multi-domain inputs separately to capture missing hits, as is the case with the primate species homologue single- and double- domain tests.

SM5.4. Table.Test 3 Compare Classification Motif set A, B and C

The following are mismatched sequences found in the third test which compare different classification motifs. The reference input A3 sequence domain file type is "both" (list of all A3 sequences) from the species clade Laurasiatheria. List of Fh1-2 sequences show amino acid differences in sequence outputs.

Seq key	species	Found in test result	Missing in test result
Li1	*Lonchorhina inusitata*	Laurasiatheria-Both-C	Laurasiatheria-Both-A
Li1	*Lonchorhina inusitata*	Laurasiatheria-Both-C	Laurasiatheria-Both-B
Li2	*Lonchorhina inusitata*	Laurasiatheria-Both-C	Laurasiatheria-Both-A
Li2	*Lonchorhina inusitata*	Laurasiatheria-Both-C	Laurasiatheria-Both-B
Li3	*Lonchorhina inusitata*	Laurasiatheria-Both-A	Laurasiatheria-Both-C
Li3	*Lonchorhina inusitata*	Laurasiatheria-Both-B	Laurasiatheria-Both-C
Li4	*Lonchorhina inusitata*	Laurasiatheria-Both-A	Laurasiatheria-Both-C
Li4	*Lonchorhina inusitata*	Laurasiatheria-Both-B	Laurasiatheria-Both-C
Fh1	*Furipterus horrens*	Laurasiatheria-Both-B	Laurasiatheria-Both-C
Fh2	*Furipterus horrens*	Laurasiatheria-Both-B	Laurasiatheria-Both-C

Fh sequences:
Fh1 – HAELCFLKWFEDTILSPYANYAVSWYVSWSPCSSCAEAVVKFLREHKKVKLNIFASRIYYYKYKDKQGLRHLDLAGAQVAMM
Fh2 – HAELCFLQWFEHTILSPYANYDVTWYASWSPCGSCAEAVSTFLREHKKVKLNIFASRIYYYNYKDKQGLRHLVLAGAQVAMM

As mentioned in the main text, motif definition is the most critical input for the algorithm. To avoid underrepresentation of genes while also reducing manual work, the motif filtering and labeling rules are set by the user which provides more flexibility as well as precision. To ensure that the motif set C test hits include all the sequences from the corresponding motif set A and B tests, we limit our pairwise analysis to the results from combined single- and double-domain homologues from the Laurasiatheria test because the same anomalies resulted in the bat and primate tests.

The motif set C results contained all the sequences from the motif set A and B tests, except for 4 sequences; Li3 and Li4 from above (in motif set A and B), and two sequences in *Furipterus horrens,* Fh1 and Fh2 (in motif set A). However, motif set C did pick up Li1 and Li2, the shorter version of Li3 and Li4 missing one of two methionine at the end (**Error! Reference source not found.** Li sequence list). This is because Z2/Z2A defined in motif set C reduced the number of uncharacterized amino acids at the end of the motif. Fh1 and Fh2 correspond to Z2B defined by motif set B but does not match the Z2B motif in set C. This is because of the change from motif set B to C where Z2B has 10 uncharacterized amino acids after the Arginine-Isoleucine-Tyrosine triplet, before the Leucine (RIY-L) instead of 9 (**Error!**

Reference source not found. Fh sequence list). Since Fh1 and Fh2 have only 9 amino acids between RIY and L the motif set B test picked them up. Out of all 27 tests, Fh1 and Fh2 are the only sequences missing completely from Laurasiatheria motif set C test. We added these manually to the final gene list table.

SM6 Scipio fine tunes nucleotide sequence ends

The Scipio track in our pipeline is optional, as it is meant to correct the start and end locations lost during the ExTRaCT track, however it is a helpful step to gain further insight into the sequence evolution. For example, sequence distances between genes can inform spatial mechanisms in certain evolutionary dynamics. Scipio takes a protein sequence and scans the entire genome assembly to find the corresponding nucleotide sequence. Our algorithm returns the first Scipio match to the final table so if sequences are duplicated in one species, one must look for additional sequence ends in the respective bed files. To run Scipio on the 102 bat genomes and 496 sequences we batched the sequence runs into 14 groups, each containing six or seven species. Running each batch in parallel took approximately 2 hours. To synthesize or characterize a gene, accurate sequence ends are required, hence the addition of this to our overall pipeline.

SM7 Validation testing using known bat A3s

To ensure that our algorithm finds true A3s, and identify false negatives, we count the number of known Z-domains from the six bat species in Jebb et al. that were found using ExTRaCT. Motif set C tests returned all the 25 known Z-domains across all clade and domain type tests, except for the double-domain primate A3 homologues test. The two missing Z-domains are both second domains and both Z3s, in the *Rhinolophus ferrumequinum* A3B (Rhifer A3B), and *Rousettus aegyptiacus* A3 (Roueag A3) genes. These two Z3 domains were found in the other Primate motif C tests. This is because none of the double domain primate A3s have a Z3 domain, the only Z3 is in the single domain human A3H, so that the HMMER profiles for double-domain primate A3s will miss homologous similar in structure to Rhifer A3B and Rouaeg A3. Motif set A and B tests had the same results between the two classification techniques, with a high rate of false negatives (22/25) and only 3 matching Z-domains to known bat A3s.

Overall, clade did not impact A3 Z-domain results, and choosing single- or double-domain homologues was only an issue in the primate results. Therefore, we can use ExTRaCT to find accurate A3 Z-domains, with 0 false negatives, if the correct labeling definitions are provided, which is why we proceed with the combined Laurasiatheria A3 motif C results. In the final gene list, we note that among the six bat species (Jebb et al., 2020) an additional eight sequences were found, different from the 25 known bat A3 Z-domains. These may be A3 paralogs or orthologs, depending on when or how the genes diverged. The sequences may be copies from duplication events or speciation events, and they may be non-coding sequences or nonfunctional genes. Species-gene reconciliation can infer whether these genes were separated by speciation (orthologs) or by duplications (paralogs) and further molecular experiments could determine functionality. Importantly, this shows that ExTRaCT finds gene family members that may be missed by conventional annotation methods.

SM7.1. Table. Count of known A3 count hits of the six bats

Count of known 6 bat A3s found by ExTRaCT categorized by input species homologue, domain structure and labeling method.

Hmmer Profile	Domain type	Salter 2016 F1 (HS)	Hayward 2018 F1 (PA)	Jebb 2020 F3 (6B)
Bat	Single	3	3	25
	Double	3	3	25
	Both	3	3	25
Primate	Single	3	3	25
	Double	3	3	23
	Both	3	3	25
Laurasiatheria	Single	3	3	25
	Double	3	3	25
	Both	3	3	25

SM8 Final gene list for phylogenetic analysis

Table by species of all Z domain counts found with ExTRaCT. This list was used

Species	Z1	Z2	Z3	Total
Antrozous pallidus	3	2	0	5
Artibeus intermedius	2	5	0	7
Artibeus lituratus	2	2	0	4
Aselliscus stoliczkanus	1	1	1	3
Brachyphylla cavernarum	1	3	0	4
Carollia perspicillata	1	3	0	4
Centurio senex	1	2	0	3
Choeroniscus minor	1	3	0	4
Cistugo seabrae	2	1	0	3
Corynorhinus mexicanus	2	2	0	4
Corynorhinus townsendii	2	2	0	4
Craseonycteris thonglongyai	1	1	1	3
Cynopterus sphinx	7	2	1	10
Desmodus rotundus	1	2	0	3
Diaemus youngii	2	2	0	4
Diphylla ecaudata	1	2	0	3
Doryrhina cyclops	2	1	1	4
Ectophylla alba	1	0	0	1
Eonycteris spelaea	3	2	1	6
Eptesicus fuscus	0	2	0	2
Eptesicus nilssonii	1	3	0	4
Erophylla bombifrons	2	3	0	5
Eumops nanus	0	1	1	2
Furipterus horrens	1	3	0	4
Glossophaga mutica	1	4	0	5
Glossophaga soricina	1	4	0	5
Glyphonycteris daviesi	1	2	0	3
Hipposideros abae	0	4	1	5
Hipposideros armiger	0	2	1	3
Hipposideros caffer	1	4	1	6
Hipposideros jonesi	0	1	1	2
Hipposideros larvatus	0	2	1	3
Hipposideros swinhoii	1	2	1	4
Hypsignathus monstrosus	0	2	1	3
Lasiurus ega	2	1	0	3
Leptonycteris yerbabuenae	1	4	0	5
Lionycteris spurrelli	1	2	0	3
Lonchorhina inusitata	1	5	0	6
Macrophyllum macrophyllum	1	4	0	5
Macrotus waterhousii	1	4	0	5
Megaderma spasma	1	2	1	4
Micronycteris megalotis	3	4	0	7
Miniopterus australis	1	1	0	2
Miniopterus natalensis	1	2	0	3
Miniopterus schreibersii	1	3	0	4
Molossus alvarezi	1	2	1	4
Molossus molossus	1	2	1	4
Molossus nigricans	1	2	1	4
Mops condylurus	1	2	1	4
Mormoops megalophylla	0	1	0	1
Myotis auriculus	3	3	0	6
Myotis californicus	0	1	0	1
Myotis daubentonii	3	5	0	8
Myotis evotis	2	4	0	6
Myotis lucifugus	16	7	0	23
Myotis myotis	1	7	0	8
Myotis mystacinus	3	3	0	6
Myotis nigricans	7	4	0	11
Myotis occultus	12	2	0	14
Myotis pilosus	4	2	0	6
Myotis velifer	8	4	0	12
Myotis vivesi	3	2	0	5
Myotis volans	1	3	0	4
Myotis yumanensis	14	4	0	18
Mystacina tuberculata	2	2	0	4

Myzopoda aurita	0	1	1	2
Natalus tumidirostris	1	1	1	3
Noctilio leporinus	1	2	0	3
Nyctalus aviator	4	2	0	6
Nycteris thebaica	1	2	2	5
Phyllostomus discolor	2	4	0	6
Phyllostomus hastatus	3	9	0	12
Pipistrellus kuhlii	5	2	0	7
Pipistrellus nathusii	2	3	0	5
Pipistrellus pygmaeus	3	5	0	8
Platyrrhinus guianensis	2	3	0	5
Plecotus auritus	1	2	0	3
Rhinolophus affinis	1	1	1	3
Rhinolophus ferrumequinum	2	2	1	5
Rhinolophus foetidus	1	2	1	4
Rhinolophus hipposideros	0	2	1	3
Rhinolophus pearsonii	2	1	1	4
Rhinolophus perniger lanosus	0	2	1	3
Rhinolophus sinicus	2	1	1	4
Rhinolophus trifoliatus	0	2	1	3
Rhinolophus yonghoiseni	0	2	1	3
Rhinophylla pumilio	1	4	0	5
Rhinopoma microphyllum	0	1	1	2
Rhinopoma muscatellum	0	1	1	2
Rhynchonycteris naso	2	2	1	5

Rousettus aegyptiacus	0	2	1	3
Saccopteryx bilineata	3	1	1	5
Saccopteryx leptura	0	1	1	2
Tadarida brasiliensis	1	2	1	4
Taphozous melanopogon	1	1	0	2
Thyroptera tricolor	1	6	0	7
Trachops cirrhosus	1	2	0	3
Triaenops persicus	0	1	1	2
Trinycteris nicefori	1	6	0	7
Uroderma convexum	2	5	0	7
Vampyressa thyone	2	6	0	8
Vespertilio murinus	3	3	0	6
TOTAL				49 8

SM9 **MSA for all Z-domains with misclassified sequence (Nt1)**

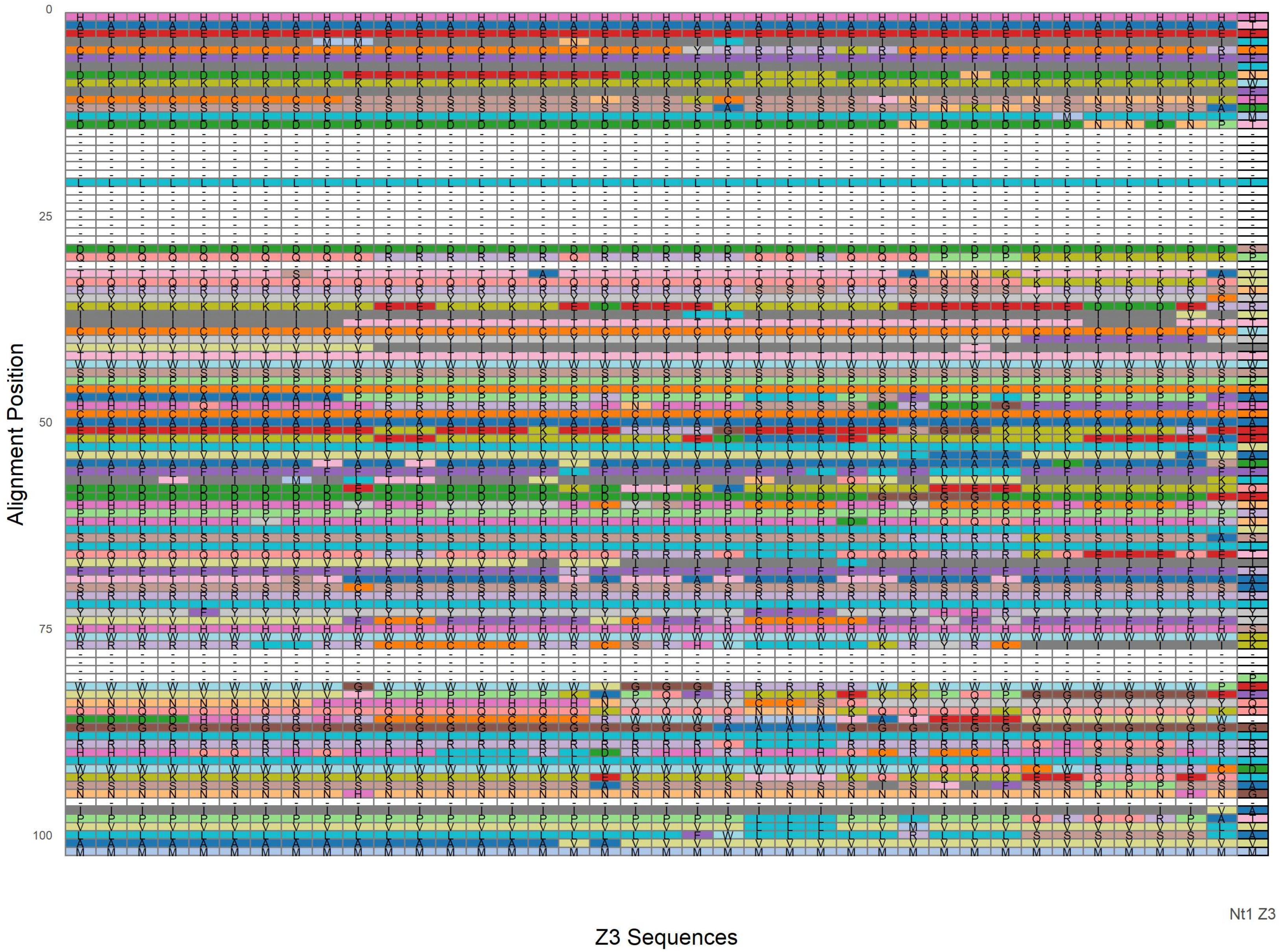

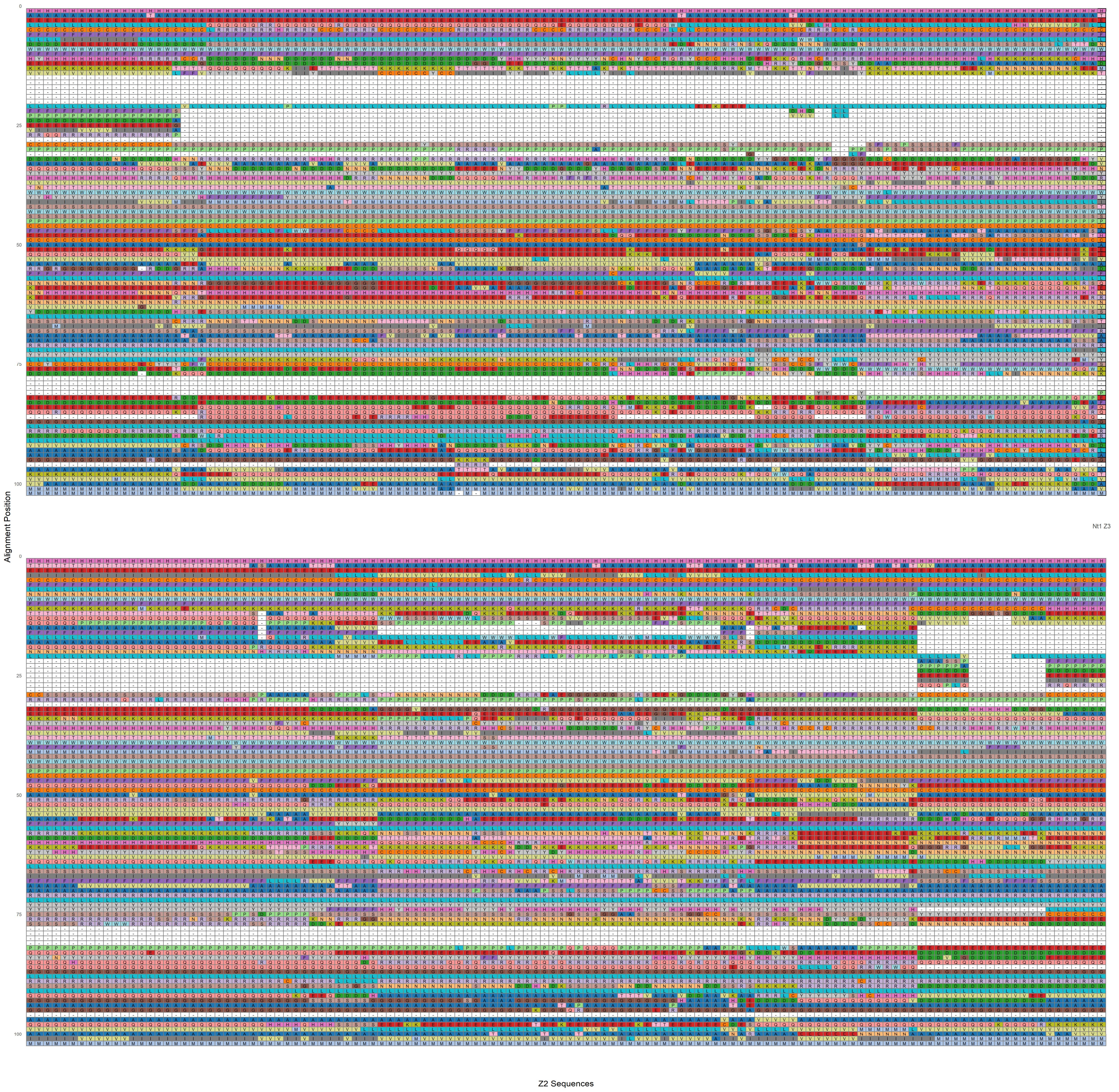

Alignment Position
0
25
50
75
100
Nt1 Z3
Z2 Sequences

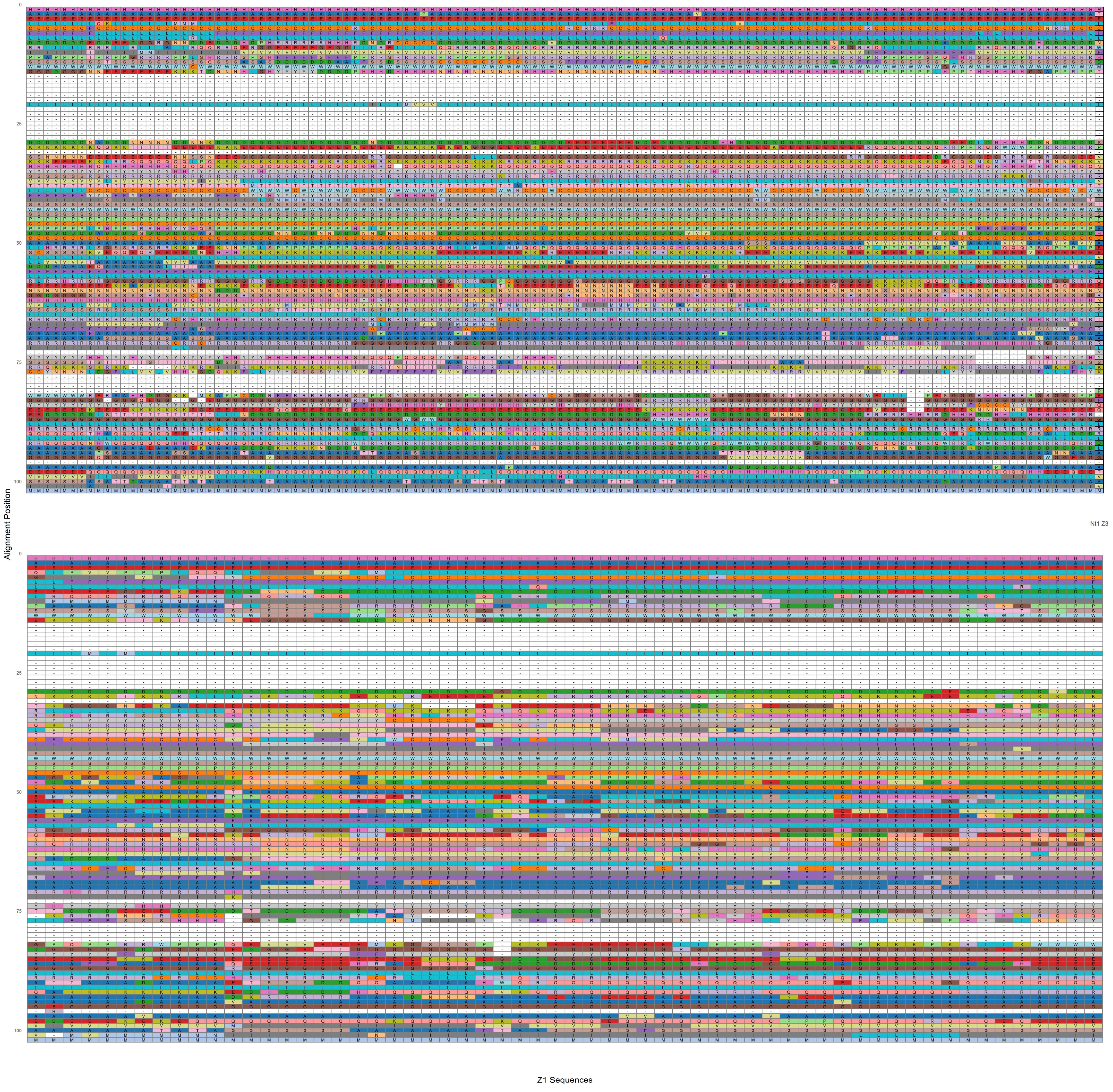
Alignment Position
0
25
50
75
100
Nt1 Z3
Z1 Sequences

SM10 References

Banerjee, A., Baker, M. L., Kulcsar, K., Misra, V., Plowright, R., & Mossman, K. (2020). Novel Insights Into Immune Systems of Bats. *Front Immunol*, *11*, 26. https://doi.org/10.3389/fimmu.2020.00026

Bat1K 21 Family Group. (2026). *Bat1K 21 Family Group* Zenodo. https://doi.org/https://doi.org/10.5281/zenodo.19001722

Becker, D. J., Eby, P., Madden, W., Peel, A. J., & Plowright, R. K. (2023). Ecological conditions predict the intensity of Hendra virus excretion over space and time from bat reservoir hosts. *Ecol Lett*, *26*(1), 23-36. https://doi.org/10.1111/ele.14007

Carlson, C. J., Brookson, C. B., Becker, D. J., Cummings, C. A., Gibb, R., Halliday, F. W., Heckley, A. M., Huang, Z. Y. X., Lavelle, T., Robertson, H., Vicente-Santos, A., Weets, C. M., & Poisot, T. (2025). Pathogens and planetary change. *Nature Reviews Biodiversity*, *1*(1), 32-49. https://doi.org/10.1038/s44358-024-00005-w

Cock, P. J., Antao, T., Chang, J. T., Chapman, B. A., Cox, C. J., Dalke, A., Friedberg, I., Hamelryck, T., Kauff, F., Wilczynski, B., & de Hoon, M. J. (2009). Biopython: freely available Python tools for computational molecular biology and bioinformatics. *Bioinformatics*, *25*(11), 1422-1423. https://doi.org/10.1093/bioinformatics/btp163

Dale, R. K., Pedersen, B. S., & Quinlan, A. R. (2011). Pybedtools: a flexible Python library for manipulating genomic datasets and annotations. *Bioinformatics*, *27*(24), 3423-3424. https://doi.org/10.1093/bioinformatics/btr539

Eby, P., Peel, A. J., Hoegh, A., Madden, W., Giles, J. R., Hudson, P. J., & Plowright, R. K. (2023). Pathogen spillover driven by rapid changes in bat ecology. *Nature*, *613*(7943), 340-344. https://doi.org/10.1038/s41586-022-05506-2

Eddy, S. R. (2011). Accelerated Profile HMM Searches. *PLoS Comput Biol*, *7*(10), e1002195. https://doi.org/10.1371/journal.pcbi.1002195

Foundation, P. S. (2021). *Python Programming Language*. In (Version 3.7.10) Python Software Foundation. https://www.python.org/downloads/release/python-3710/

Hayward, J. A., Tachedjian, M., Cui, J., Cheng, A. Z., Johnson, A., Baker, M. L., Harris, R. S., Wang, L. F., & Tachedjian, G. (2018). Differential Evolution of Antiretroviral Restriction Factors in Pteropid Bats as Revealed by APOBEC3 Gene Complexity. *Mol Biol Evol*, *35*(7), 1626-1637. https://doi.org/10.1093/molbev/msy048

Hoyt, B. (2024). *regex*. In (Version 2024.4.16) Python Package Index (PyPI). https://pypi.org/project/regex/

Jebb, D., Huang, Z., Pippel, M., Hughes, G. M., Lavrichenko, K., Devanna, P., Winkler, S., Jermiin, L. S., Skirmuntt, E. C., Katzourakis, A., Burkitt-Gray, L., Ray, D. A., Sullivan, K. A. M., Roscito, J. G., Kirilenko, B. M., Davalos, L. M., Corthals, A. P., Power, M. L., Jones, G., . . . Teeling, E. C. (2020). Six reference-quality genomes reveal evolution of bat adaptations. *Nature*, *583*(7817), 578-584. https://doi.org/10.1038/s41586-020-2486-3

Katoh, K., & Standley, D. M. (2013). MAFFT multiple sequence alignment software version 7: improvements in performance and usability. *Mol Biol Evol*, *30*(4), 772-780. https://doi.org/10.1093/molbev/mst010

Keller, O., Odronitz, F., Stanke, M., Kollmar, M., & Waack, S. (2008). Scipio: using protein sequences to determine the precise exon/intron structures of genes and their orthologs in closely related species. *BMC Bioinformatics*, *9*, 278. https://doi.org/10.1186/1471-2105-9-278

NCBI Resource Coordinators. (2016). Database resources of the National Center for Biotechnology Information. *Nucleic Acids Res*, *44*(D1), D7-19. https://doi.org/10.1093/nar/gkv1290

O'Shea, T. J., Cryan, P. M., Cunningham, A. A., Fooks, A. R., Hayman, D. T., Luis, A. D., Peel, A. J., Plowright, R. K., & Wood, J. L. (2014). Bat flight and zoonotic viruses. *Emerg Infect Dis*, *20*(5), 741-745. https://doi.org/10.3201/eid2005.130539

Perez, G., Barber, G. P., Benet-Pages, A., Casper, J., Clawson, H., Diekhans, M., Fischer, C., Gonzalez, J. N., Hinrichs, A. S., Lee, C. M., Nassar, L. R., Raney, B. J., Speir, M. L., van Baren, M. J., Vaske, C. J., Haussler, D., Kent, W. J., & Haeussler, M. (2025). The UCSC Genome Browser database: 2025 update. *Nucleic Acids Res*, *53*(D1), D1243-D1249. https://doi.org/10.1093/nar/gkae974

Reback, J., jbrockmendel, McKinney, W., Van den Bossche, J., Augspurger, T., Cloud, P., Hawkins, S., Roeschke, M., gfyoung, Sinhrks, Klein, A., Petersen, T., Hoefler, P., Tratner, J., She, C., Ayd, W., Naveh, S., Garcia, M., Darbyshire, J., & Seabold, S. (2021). *pandas-dev/pandas: Pandas 1.3.5 (v1.3.5)*. In

Rice, P., Longden, I., & Bleasby, A. (2000). EMBOSS: the European Molecular Biology Open Software Suite. *Trends Genet*, *16*(6), 276-277. https://doi.org/10.1016/s0168-9525(00)02024-2

Salter, J. D., Bennett, R. P., & Smith, H. C. (2016). The APOBEC Protein Family: United by Structure, Divergent in Function. *Trends Biochem Sci*, *41*(7), 578-594. https://doi.org/10.1016/j.tibs.2016.05.001

Scipio, T. (2025). *Scipio eukaryotic gene identification*. In https://www.webscipio.org/help/scipio#requirements

Stamatakis, A. (2014). RAxML version 8: a tool for phylogenetic analysis and post-analysis of large phylogenies. *Bioinformatics*, *30*(9), 1312-1313. https://doi.org/10.1093/bioinformatics/btu033

Stanke, M., & Morgenstern, B. (2005). AUGUSTUS: a web server for gene prediction in eukaryotes that allows user-defined constraints. *Nucleic Acids Res*, *33*(Web Server issue), W465-467. https://doi.org/10.1093/nar/gki458

UCSC. (2025). *Senckenberg Genome Browser*. https://genome.senckenberg.de/

Zhang, G., Cowled, C., Shi, Z., Huang, Z., Bishop-Lilly, K. A., Fang, X., Wynne, J. W., Xiong, Z., Baker, M. L., Zhao, W., Tachedjian, M., Zhu, Y., Zhou, P., Jiang, X., Ng, J., Yang, L., Wu, L., Xiao, J., Feng, Y., . . . Wang, J. (2013). Comparative analysis of bat genomes provides insight into the evolution of flight and immunity. *Science*, *339*(6118), 456-460. https://doi.org/10.1126/science.1230835
